# Supplementary material for: Palmprint recognition based on principal line features
Source: PeerJ Comput Sci. 2025 Aug 18;11:e3109. doi: 10.7717/peerj-cs.3109 (PMC12453761; doi:10.7717/peerj-cs.3109)

Here are some typical examples of palmprint samples.


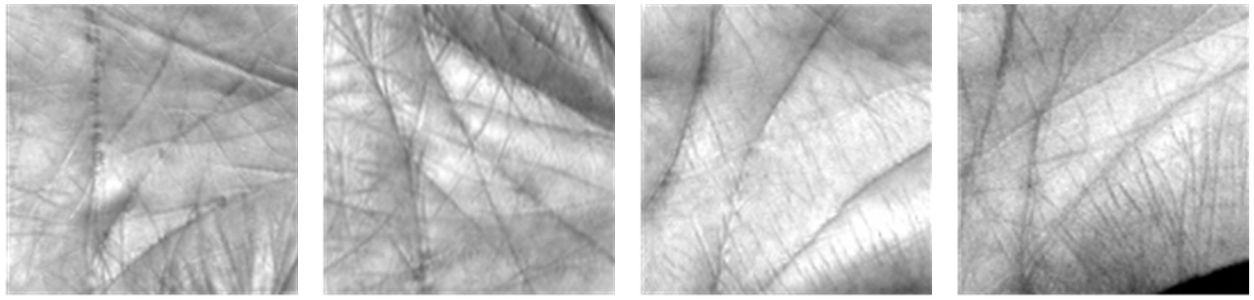


Fig. 1 respectively show four palmprint ROI images from different palms in PolyU II database.


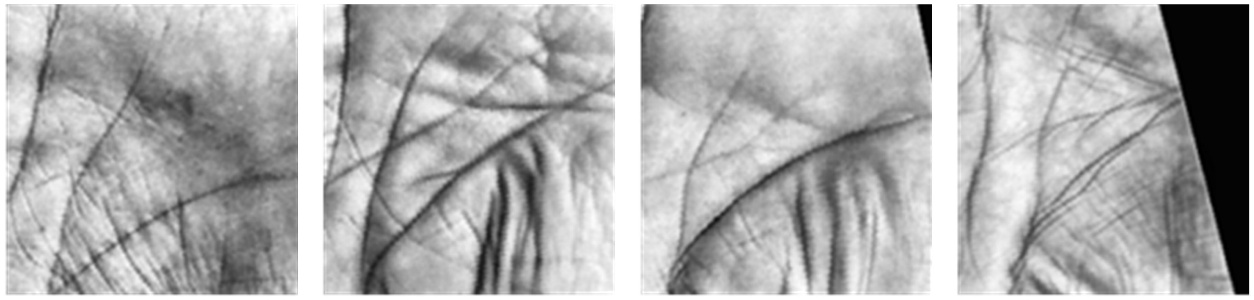


Fig. 2 respectively show four palmprint ROI images from different palms in IIT Delhi database.


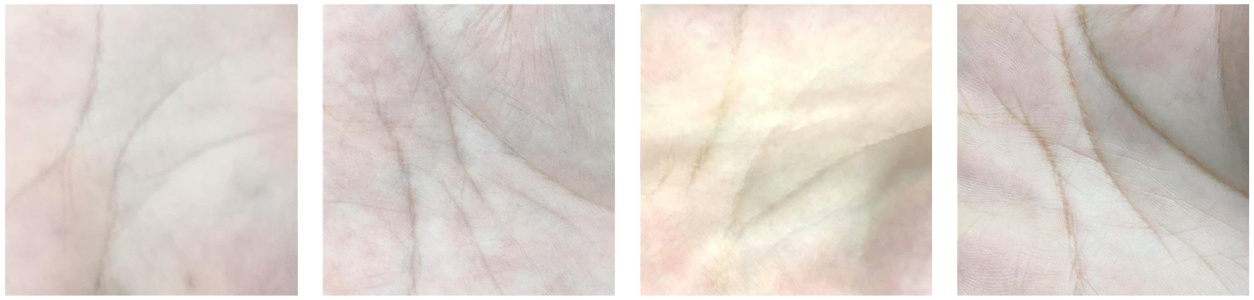
Fig. 3 respectively show four palmprint ROI images from different palms in XINHUA database.


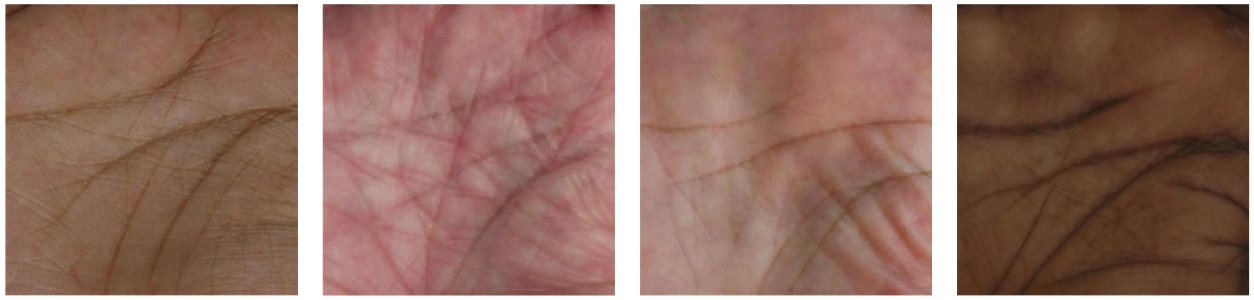
Fig. 4 respectively show four palmprint ROI images from different palms in NTU-CP-V1 database.


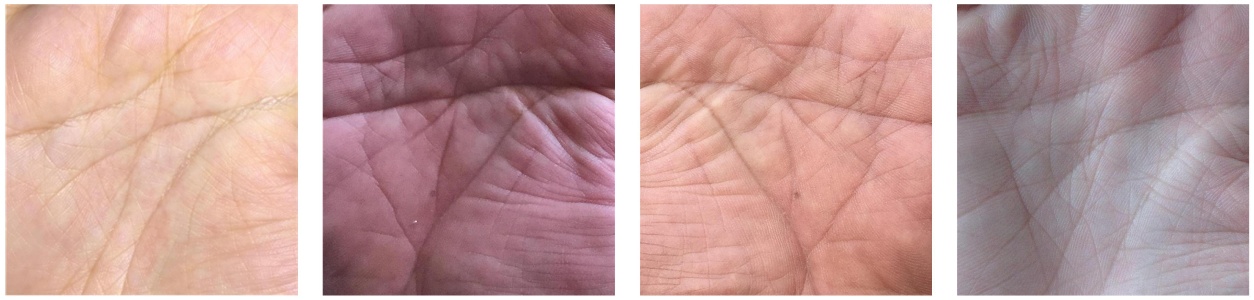


Fig. 5 respectively show four palmprint ROI images from different palms in BJTU-CP-V2 database.


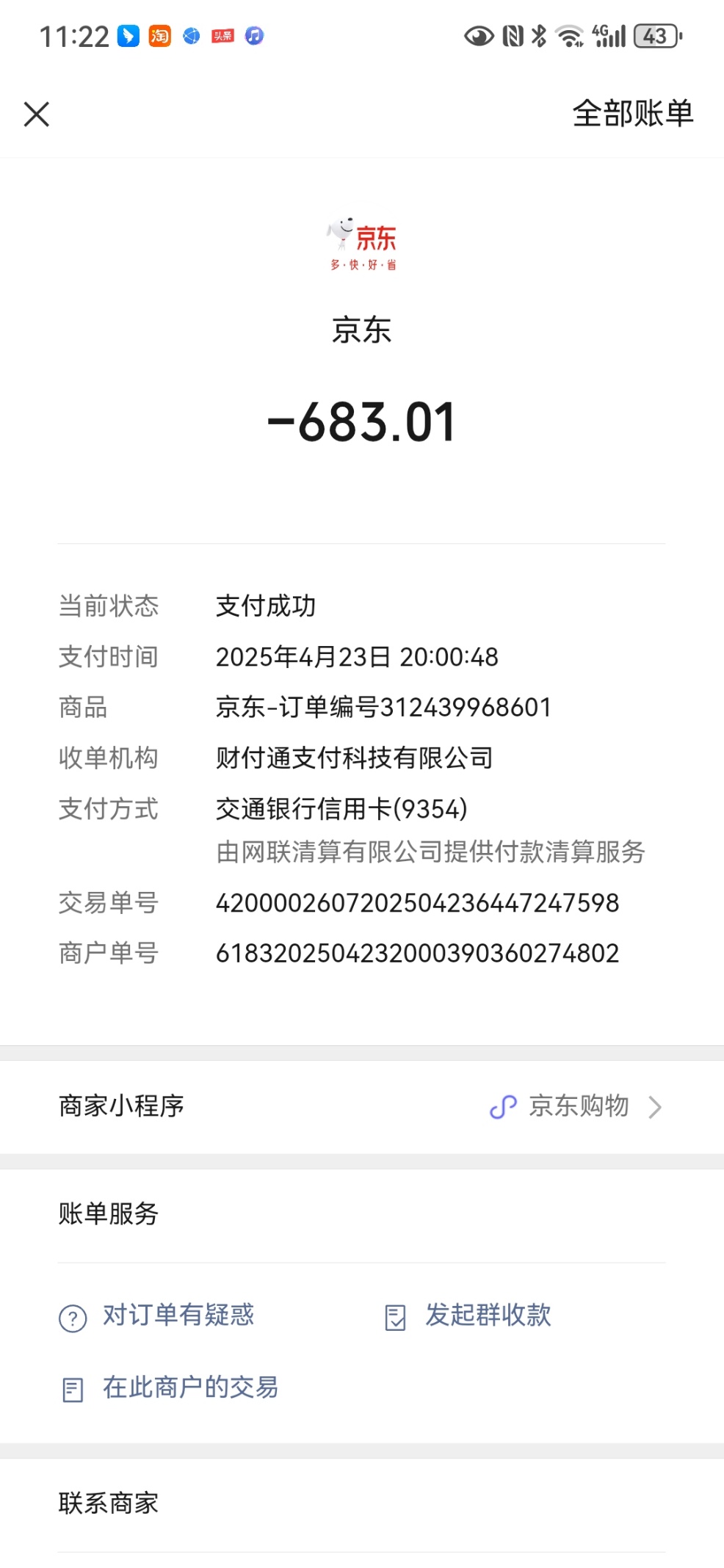

Supplement: Supplemental Information 2 [file peerj-cs-11-3109-s002.docx]
