# Supplementary figures and images for: Palmprint recognition based on principal line features
Source: PeerJ Comput Sci. 2025 Aug 18;11:e3109. doi: 10.7717/peerj-cs.3109 (PMC12453761; doi:10.7717/peerj-cs.3109)

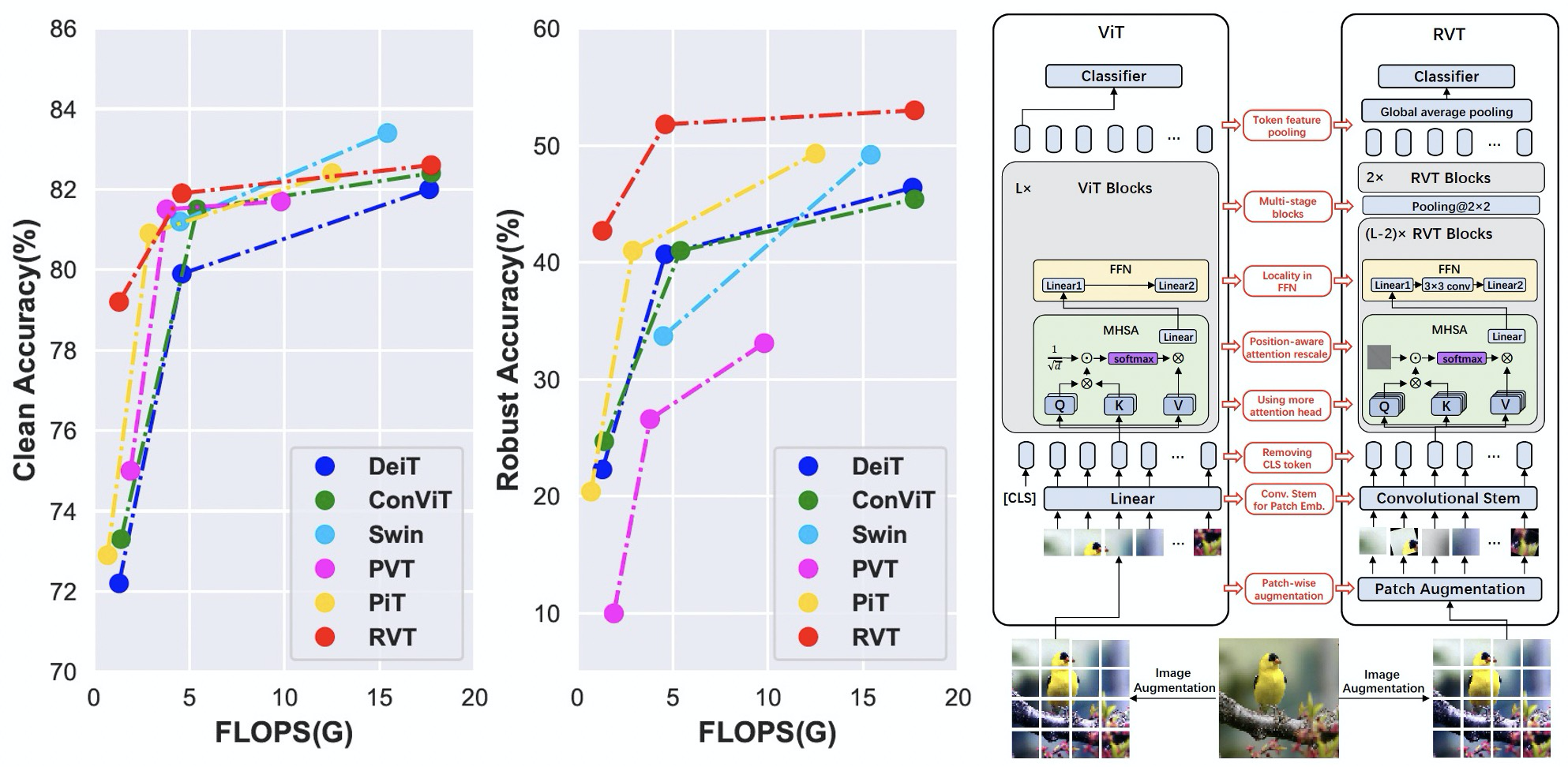

Supplement: Supplemental Information 3 [file peerj-cs-11-3109-s003.zip › LViT/models/RVT/RVT.png]
