# Supplementary material for: Palmprint recognition based on principal line features
Source: PeerJ Comput Sci. 2025 Aug 18;11:e3109. doi: 10.7717/peerj-cs.3109 (PMC12453761; doi:10.7717/peerj-cs.3109)
Supplement: Supplemental Information 4 [file peerj-cs-11-3109-s004.docx]

## 3D Palmprint experiment

Although this study focuses on 2D palmprint recognition, the development of 3D palmprint has great potential and broad prospects. 3D palmprint covers a wealth of information about the three-dimensional surface of the palm, including unique undulating and concave-convex details, which is richer than 2D palmprint features, greatly improves the recognition accuracy and reliability, and effectively reduces the false recognition rate and rejection rate. In terms of anti-counterfeiting, its complex three-dimensional structure is extremely difficult to forge, and its security is much higher than that of 2D palm prints, which is suitable for high security demand scenarios such as finance and military. At the same time, the 3D palmprint has stable features under different illumination and shooting angles, less external interference and stronger adaptability. Moreover, each person's 3D palmprint is unique, and identical twins also have subtle differences, which are extremely unique and can accurately distinguish individuals. Recent research results fully show the great potential and application value of 3D palmprint recognition(Fei et al., 2020; Sen Lin et al., 2024; Su et al., 2025).

In this section, experiments are carried out in the open palm print data set PolyU 3D(Li et al., 2011) which is a contact 3D palm print data set. The database collects 400 palms of 200 people's left and right hands. The collection stage is divided into two stages, with an interval of one month. Ten palm print images are collected in each stage, and 8000 palm print images are finally obtained.

Generally, 3D palmprint data preserves the depth information of a palm surface. The

original captured 3D palmprint data is a small positive or negative float, which is usually transformed into the grey level value for practical feature extraction. To facilitate the design of recognition algorithms, the original 3D palmprint data is usually transformed into a curvature-based data. Two most important curvatures include the mean curvature (MC) and gaussian curvature (GC), and their corresponding images are mean curvature image (MCI) and gaussian

curvature image (GCI). Based on GC and MC, two new grey-level image representations have been proposed including surface type (ST) and compact ST (CST). Since the representations of MCI, GCI, ST and CST depict a 3D palmprint as a 2D grey-level palmprint image, those 2D palmprint recognition methods can be used for 3D palmprint recognition. Fig. 1 shows the examples of original 3D palmprint, 3D palmprint ROI, and four 2D representations including MCI, GCI, ST and CST. This paper will use four 2D representations of 3D palmprint to carry out experiments.

Table 1 is the result of 3D palmprint experiment. From the table, we can see that L-ConvMixer has achieved the best results on all data sets except MCI data set, which also proves the robustness of LViT paradigm. In addition, depth models such as EEPNet, CCNet, and CO3Net perform poorly on 3D data sets, which may be that global curvature features are more important than local curvature features. Compared with CNN, Transform architecture shows unique advantages in processing 3D palmprint curvature images. CNN captures local features through convolution operation, and performs well in extracting subtle texture changes, but it has limitations in integrating global curvature features. Its receptive field is limited by the size of convolution kernel, so it is difficult to obtain the whole curvature distribution of palm print at one time. The Transform architecture is based on the self-attention mechanism, which can calculate the correlation between each position and all other positions in the image in parallel, and can directly establish the dependency relationship in the global range, which makes it more efficient to aggregate the global curvature features. In addition, the Transform architecture also has stronger long-distance dependency modeling ability, and can effectively capture the relationship between the regions that are far apart in the 3D palmprint but are related to the global curvature features, thus achieving more accurate judgment in the image recognition task based on the global curvature features.


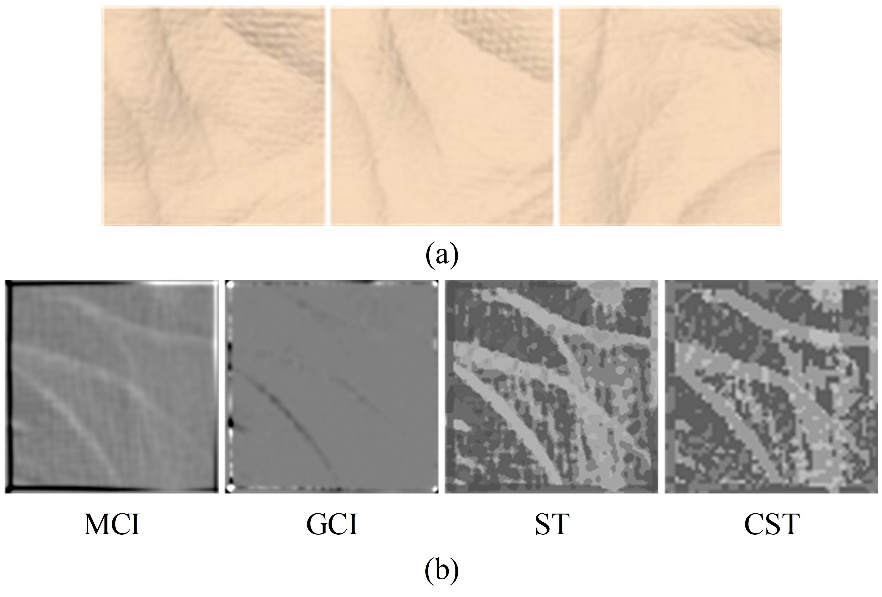


**Figure 1** PolyU 3D Palmprint ROI and Its Four 2D Manifestations: (a)Three ROI images of 3D palmprint (b)Four 2D Manifestations of 3D Palmprint ROI.

Table 1 3D palmprint experiment results.

| **Methods** | **MCI** | | **GCI** | | **ST** | | **CST** | |
| --- | --- | --- | --- | --- | --- | --- | --- | --- |
|  | **ARR** | **EER** | **ARR** | **EER** | **ARR** | **EER** | **ARR** | **EER** |
| CompC (Zhang et al., 2003) | 97.61 | 2.1564 | 72.64 | 18.6315 | 99.47 | 0.3847 | 99.17 | 0.5020 |
| OrdinalC (Sun et al., 2005) | 97.86 | 1.4220 | 97.97 | 1.4462 | 99.81 | 0.1659 | 99.75 | 0.3388 |
| RLOC (Jia et al., 2008) | 99.33 | 0.7187 | 96.89 | 1.7188 | 99.83 | 0.2819 | 99.31 | 0.4036 |
| LLDP (Luo et al., 2016) | 95.84 | 2.4376 | 94.58 | 2.7588 | 98.14 | 0.8679 | 95.86 | 1.9521 |
| EEPNet (Jia et al., 2022) | 94.63 | 2.5672 | 92.84 | 3.2728 | 96.68 | 2.0215 | 97.05 | 1.9732 |
| CCNet (Yang, et al., 2023) | 94.22 | 2.8535 | 93.42 | 3.0572 | 96.55 | 2.1516 | 98.87 | 0.7525 |
| CO3Net (Yang, et al., 2023) | 94.52 | 2.6326 | 91.47 | 4.4043 | 94.65 | 2.6244 | 97.47 | 1.8532 |
| L-ViT (Proposed) | 97.36 | 1.4521 | 96.77 | 1.9896 | 98.47 | 0.7854 | 98.44 | 0.8846 |
| L-Conformer (Proposed) | 96.16 | 1.8626 | 94.56 | 2.8356 | 96.19 | 2.4335 | 95.38 | 2.3325 |
| L-PVT-V2 (Proposed) | 97.43 | 1.3392 | 97.93 | 1.5348 | 97.45 | 1.9024 | 98.79 | 0.8248 |
| L-ConvMixer (Proposed) | 98.13 | 0.8524 | **98.75** | **0.7813** | **99.87** | **0.1276** | **99.85** | **0.2328** |
